# Editorial: Deciphering dopamine dysregulation in adult ADHD

**DOI:** 10.1002/pcn5.70151

**Published:** 2025-06-29

**Authors:** Norio Yasui‐Furukori

**Affiliations:** ^1^ Department of Psychiatry, School of Medicine Dokkyo Medical University Mibu Japan

The study by Itagaki et al.,^1^ recently published in *Psychiatry and Clinical Neurosciences Reports*, has been honored with the prestigious 2024 PCNR Award in recognition of its outstanding contribution to the field of neuropsychiatric research. This accolade underscores the study's significant impact in advancing our understanding of attention‐deficit/hyperactivity disorder (ADHD) through imaging techniques. While previous research has struggled with methodological inconsistencies, this study provides a valuable foundation for future investigations into the neurobiological underpinnings of ADHD.

Rather than focusing solely on the study's specific findings, it is essential to highlight its broader implications for the future of ADHD neuroimaging. The field of psychiatric imaging has evolved significantly in recent years, with advances in positron emission tomography (PET), single‐photon emission computed tomography (SPECT), and functional magnetic resonance imaging (fMRI) offering unprecedented insights into the neurochemical and structural abnormalities associated with ADHD. The study by Itagaki et al.^1^ exemplifies the potential of these technologies to unravel complex neurobiological processes. One of the most promising directions in ADHD imaging research is the integration of multimodal approaches. Combining PET and fMRI, for instance, could enable researchers to examine both neurotransmitter function and network connectivity within the brain. Moreover, machine learning algorithms applied to large‐scale imaging datasets hold the potential to identify novel biomarkers for ADHD, improving both diagnostic precision and treatment stratification. As imaging techniques continue to advance, future studies may refine our understanding of how ADHD subtypes differ neurobiologically, paving the way for more personalized interventions.

The study by Itagaki et al.^1^ adds to a growing body of research that seeks to understand the role of dopamine dysregulation in ADHD. The notion that ADHD involves abnormalities in dopamine transporter (DAT) function was initially highlighted by Wang et al.,^2^ who observed increased DAT availability following chronic stimulant treatment, suggesting that previous imaging studies may have been confounded by medication effects. However, attempts to quantify DAT availability in ADHD patients have yielded mixed results, as noted in a meta‐analysis by Fusar‐Poli et al.,^3^ which attributed these discrepancies to variations in medication status and imaging methodologies. Addressing this concern, Itagaki et al.^1^ focused exclusively on drug‐naive adults, thereby providing a clearer perspective on DAT function in ADHD. Further supporting this, Volkow et al.^4^ used PET imaging to demonstrate altered dopamine reward pathways in adults with ADHD, providing a foundation for the current understanding of motivational deficits. Beyond DAT imaging, research by Plichta and Scheres^5^ using fMRI has illustrated the functional consequences of dopamine dysregulation, showing reduced ventral striatal activation during reward anticipation in ADHD patients. This aligns with the present study's emphasis on the nucleus accumbens, reinforcing the idea that deficits in reward processing are central to the disorder's pathophysiology. Lastly, Barlow et al.^6^ provided further evidence linking impulsivity to dopamine system abnormalities, specifically demonstrating a correlation between ventral striatal dopamine receptor availability and impulsive choice behavior. The findings of Itagaki et al.^1^ complement this work by showing that DAT availability is not only relevant to impulsivity but also to inattentive symptoms, particularly in relation to the caudate nucleus. Collectively, these studies illustrate a cohesive narrative: ADHD is not merely a disorder of attention, but one deeply rooted in disrupted dopaminergic function, with implications for motivation, impulsivity, and executive control.

While the study by Itagaki et al.^1^ represents a significant advancement in ADHD neuroimaging, some limitations should be acknowledged. The relatively small sample size may limit the generalizability of the findings, and the resolution of SPECT imaging, while valuable, is lower than that of PET, which may impact the precision of DAT measurements. Additionally, the study focuses solely on DAT availability, whereas other neurochemical pathways, such as dopamine receptor density and synthesis, may also play crucial roles in ADHD pathophysiology. Future research should incorporate larger sample sizes, higher‐resolution imaging techniques, and multimodal approaches to obtain a more comprehensive understanding of ADHD's neurobiological basis.

By earning the 2024 PCNR Award, Itagaki et al.^1^ have set a new benchmark for neuroimaging research in ADHD. Their study highlights the promise of advanced imaging techniques in clarifying the disorder's neurobiological basis, while also raising important questions for future research. As the field moves toward integrative, multimodal approaches, the next decade is likely to witness substantial breakthroughs in ADHD diagnosis, classification, and treatment. These advancements will not only deepen our understanding of ADHD but also enhance clinical outcomes through precision medicine approaches.

## CONFLICT OF INTEREST STATEMENT

The author declares no conflicts of interest.

## Data Availability

Research data are not shared.
